# Case Management in Primary Care Associated With Service Use by Adults With Dementia and Comorbidities

**DOI:** 10.1093/geroni/igab046.1287

**Published:** 2021-12-17

**Authors:** Emma Quach, Lauren Moo, Christine Hartmann, Shibei Zhao, Pengsheng Ni

**Affiliations:** 1 Center for Healthcare Organization and Implementation Research, VA Bedford Healthcare System, Massachusetts, United States; 2 VA Bedford Health Care System, Bedford, Massachusetts, United States; 3 VA Bedford Healthcare System, VA Bedford Healthcare System, Massachusetts, United States; 4 VA Bedford Healthcare System, Bedford, Massachusetts, United States; 5 Boston University School of Public Health, Boston University School of Public Health, Massachusetts, United States

## Abstract

Community-dwelling adults with dementia are at higher risks than counterpart without dementia of poor health outcomes, and those with dementia and co-occurring conditions face even greater risks. Optimal treatment for dementia includes functional and psychosocial support through long-term services and supports (LTSS), but use remains low. Our study investigated whether case management provided in primary care and in dementia care settings facilitated LTSS use for Veterans with dementia and comorbidities. We performed a cross-sectional analysis of 2019 VA-paid health care on a cohort of Veterans with dementia, defined by clinical diagnoses (International Classification of Disease, Tenth Revision). Receipt of case management was measured by whether or not a Veteran enrolled in a VA (1) home-based primary care, (2) geriatric primary care, or (3) dementia clinic. Comorbidities were measured by an adapted Elixhauser comorbidities index and dichotomized as ≤ 3 or ≥ 4 comorbidities. LTSS use was measured by whether or not Veterans used home health, home respite, adult day care, hospice, or veteran-directed care. Multivariate logistic regressions showed that LTSS use was higher for enrollees in each case management program compared to Veterans not enrolled in any. LTSS use was also higher for enrollees in each primary care program with more comorbidities than program counterparts with fewer comorbidities. Case management in primary care settings may facilitate functional and psychosocial support to meet dementia and non-dementia related needs for adults who have dementia with comorbidities.

